# Stimulation of Peripheral Blood Mononuclear Cells with *Lactococcus lactis* Strain Plasma Elicits Antiviral Effects Against H1N1 and SARS-CoV-2

**DOI:** 10.3390/ijms262311573

**Published:** 2025-11-28

**Authors:** Zhao Xuan Low, Owen Woo, Osamu Kanauchi, Pouya Hassandarvish, Vunjia Tiong, Sazaly AbuBakar

**Affiliations:** 1 Tropical Infectious Diseases Research and Education Centre (TIDREC), Higher Institution Centre of Excellence, Universiti Malaya, Kuala Lumpur 50603, Malaysia; lowzx95@gmail.com (Z.X.L.); owenwoo58@gmail.com (O.W.); kanauchio@kirin.co.jp (O.K.); pouyahassandarvish@um.edu.my (P.H.); 2 Institute of Health Sciences, Kirin Holdings Co., Ltd., 2-26-1, Muraoka-Higashi, Fujisawa 251-8555, Kanagawa, Japan; 3 Department of Biomedical Science, Faculty of Medicine, Universiti Malaya, Kuala Lumpur 50603, Malaysia

**Keywords:** H1N1, SARS-CoV-2, LC-Plasma, antiviral, probiotics, immunomodulation

## Abstract

Viruses, like influenza and Severe acute respiratory syndrome coronavirus 2 (SARS-CoV-2), remain major causes of upper respiratory tract infections worldwide, with symptoms ranging from asymptomatic to lethal outcomes. While antivirals and vaccines have helped ameliorate disease morbidity and mortality, these infections still pose significant challenges. Probiotics, including *Lactococcus lactis* strain plasma (LC-Plasma), have recently shown antiviral effects by activating plasmacytoid dendritic cells (pDCs), though their detailed mechanism remains unclear. In this study, we stimulated peripheral blood mononuclear cells (PBMCs) collected from healthy participants with LC-Plasma and conducted immunological analyses to investigate the immunomodulatory mechanisms of LC-Plasma. The supernatant derived from LC-Plasma-stimulated PBMCs (LCP Sup) exhibited dose-dependent inhibition of replication in Influenza A virus subtype H1N1 (H1N1) and SARS-CoV-2. LCP Sup significantly reduced the SARS-CoV-2 viral load in Huh-7 cells. However, in the H1N1 antiviral assay using A549 cells, LCP Sup was required at a higher concentration against H1N1 in A549 cells compared with SARS-CoV-2 in Huh-7 cells. Treatment with LCP Sup significantly upregulated interferon-stimulated genes (ISG) expression, particularly *MxA*, in A549 cells. While *MxA* showed the most notable increase, other ISGs also exhibited elevated expression levels compared with the negative control. Other cytokines, chemokines, and growth factors were also induced by LC-Plasma and CpG-DNA stimulation, and the effects of LC-Plasma were much higher than those of CpG-DNA. These results provide in vitro evidence of the antiviral mechanisms of LC-Plasma via upregulation of interferon-α (IFN-α) and related ISGs for host defense against respiratory viruses.

## 1. Introduction

Coronavirus disease 2019 (COVID-19), caused by severe acute respiratory syndrome coronavirus-2 (SARS-CoV-2), continues to spread globally and remains a major public health issue. Although several vaccines have been approved, containing COVID-19 remains challenging. This is due to the ongoing emergence of SARS-CoV-2 variants, such as the EG.5 variant (peaking in late 2023) and the JN.1 variant (peaking in early 2024), both of which increase transmission and evade immunity [1]. Since late 2021, the Omicron variant has dominated the pandemic [2], with many studies reporting vaccine breakthrough infections and reinfections [3]. Protection from booster doses is short-lived because neutralizing antibody levels drop quickly [4]. Frequent booster doses with a limited systemic immune response may cause immune tolerance, potentially leading to less effective antibodies and reduced activation of effector T cells [5,6]. No definitive treatment exists yet, and severe cases with high morbidity and mortality are more common in elderly individuals and those with underlying health conditions. COVID-19 often starts with non-specific upper respiratory symptoms, making it hard to distinguish from other illnesses without advanced diagnostics. In addition, influenza often causes symptoms similar to COVID-19, and limited understanding of COVID-19 can lead to misdiagnosis or missed co-infections. Human influenza A virus (IAV) causes both seasonal flu and pandemics [7], with over 130 subtypes arising due to its high mutation rate. Besides vaccines, antiviral drugs such as oseltamivir (Tamiflu), zanamivir (Relenza), and peramivir (Rapivab) target the influenza virus N protein [8]. However, rapid evolution of IAV and SARS-CoV-2, along with their ability to develop resistance to antivirals and vaccines, means the development of next-generation treatments is still needed.

*Lactococcus lactis* strain plasma (LC-Plasma), also known as *Lactococcus lactis* subsp. *lactis* JCM5805, was identified as a strain capable of significantly inducing interferon (IFN)-α production in dendritic cells (DCs) among more than 100 strains screened [9]. It was reported that LC-Plasma can be internalized and incorporated into plasmacytoid dendritic cells (pDCs), producing enhanced IFN-α in vivo [9]. This finding is particularly intriguing, as pDCs rarely internalize whole bacteria, which are relatively much larger. Our previous study demonstrated that LC-Plasma effectively stimulates human pDCs within peripheral blood mononuclear cells (PBMCs). Plasmacytoid DCs (pDCs) are a rare subset of DCs proficient at producing Type I IFNs. These cells constitute only 0.2–0.8% of the PBMC population [10]. The pDCs originate from hematopoietic stem cells (HSCs) in the bone marrow via both myeloid and lymphoid precursors [11]. Similar to other DCs, upon virus activation, pDCs can secrete cytokines and chemokines, as well as increase the expression of co-stimulatory molecules such as CD80, CD86, and CD40 to prime CD8+ and CD4+ T cells [12]. Human pDCs express elevated levels of Toll-like receptor 9 (TLR9) and efficiently retain viral genomes within the endosomes, in contrast to the conventional myeloid dendritic cells (mDCs) or DCs [13]. This retention prevents the rapid transport of viral genomes to the lysosome, allowing sufficient time for converting these viral genomes into effective TLR ligands, thus upregulating Type I IFN production [13]. Therefore, stimulation of pDCs is considered to produce a protective effect against virus infection through the stimulation and production of IFNs.

Our previous in vitro studies have shown that humoral factors derived from LC-Plasma-stimulated PBMCs when used to treat Huh-7 cells can upregulate interferon-stimulated genes (ISGs) [14]. Recently, host-directed therapy (HDT) has emerged as a promising approach in the field of infectious diseases. HDT strategies aim to interfere with the host cell factors required by pathogens for replication or persistence, enhance protective immune responses against pathogens, reduce excessive inflammation, and balance immune reactivity at pathological sites. IFN treatment for chronic viral hepatitis is a well-established example of HDT; however, novel medical options for this viral infection remain critically needed [15]. Currently, it is challenging to identify vaccines or antiviral agents with broad-spectrum efficacy against multiple viruses; existing therapies are effective only against specific viruses. If safe interventions, such as food-derived materials, could be discovered that prevent infections regardless of virus type, they could offer economically feasible and highly beneficial solutions for healthcare and human welfare, although such options are still under development [16]. The present study sought to determine whether humoral factors from LC-Plasma-stimulated PBMCs would induce ISG expression in human cells and inhibit Influenza A virus subtype H1N1 (H1N1) and SARS-CoV-2 replication.

## 2. Results

### 2.1. LC-Plasma-Stimulation of PBMCs Produce Factors That Inhibit H1N1 and SARS-CoV-2 Replication

**Figure 1:**
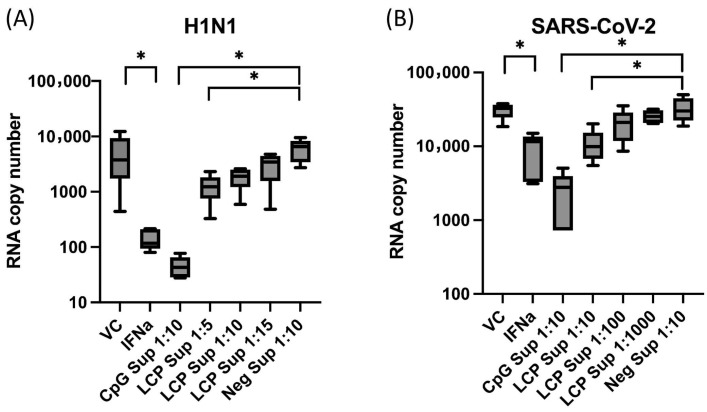
Antiviral effects of the supernatant from PBMCs stimulated with LC-Plasma against H1N1 and SARS-CoV-2 replication. Comparison of (**A**) H1N1 and (**B**) SARS-CoV-2 viral titers in treated A549 and Huh-7 cells, respectively. The experiment design contained a virus control (VC), interferon alpha (IFN-α), CpG supernatant (CpG Sup) as inducer for innate immunity, *Lactococcus lactis* strain plasma supernatant (LCP Sup) at different concentrations, and negative supernatant (Neg Sup) as normal cells. The box plots represent the median ± SD (*N* = 5). * *p* < 0.05 indicates a significant difference (non-parametric Kruskal–Wallis and Dunn’s multiple comparisons test).

Dose-dependent antiviral effects against H1N1 and SARS-CoV-2 were observed with the serially diluted LC-Plasma-stimulated PBMC supernatant (LCP Sup) (Figure 1). The results showed that IFN-α, 10-fold diluted CpG-treated PBMC supernatant (CPG Sup 1:10), and 5-fold diluted LCP Sup (1:5) significantly reduced H1N1 titers in A549 (Figure 1A). Similarly, IFN-α, CpG Sup 1:10, and LCP Sup 1:10 significantly reduced SARS-CoV-2 titers in Huh-7 cells (Figure 1B). Using the non-parametric Kruskal–Wallis and Dunn’s multiple comparisons test, the reduction in the viral RNA copy numbers of H1N1 and SARS-CoV-2 was statistically significant (*p* < 0.05).

### 2.2. Upregulation of ISGs in A549 Cells After Treatment with Supernatant from LC-Plasma-Stimulated PBMCs

**Figure 2:**
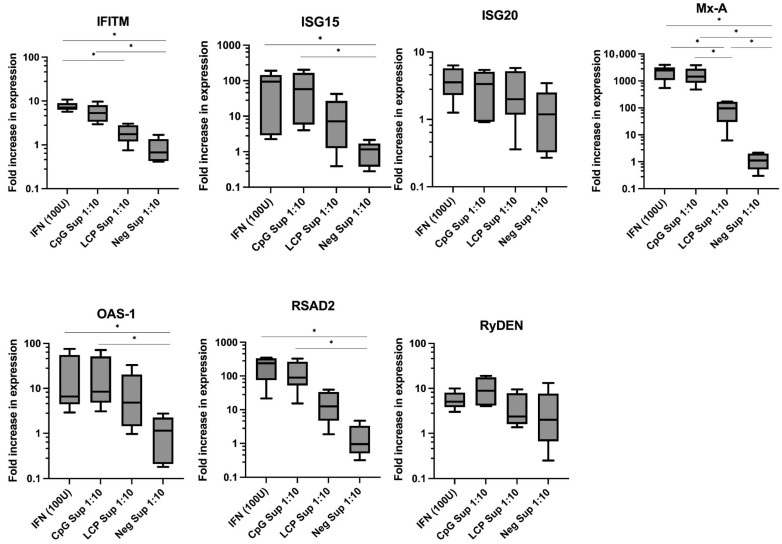
Induction of IFN-stimulated gene (ISG) expression in A549 Cells. A549 cells were exposed to recombinant IFN-α (100 units), CpG-treated Sup 1:10, LCP Sup 1:10, or Neg Sup 1:10 for 24 h, and the expression of *ISGs*, *RyDEN*, *IFITM-1*, *OAS-1*, *ISG15*, *ISG20*, *RSAD2* and *MxA* were determined. The gene expression levels were referenced to the untreated A549 cells (cell control) and normalized beta-actin gene. The experiment used interferon alpha (IFN), CpG supernatant (CpG Sup) as an inducer for innate immunity, *Lactococcus lactis* strain plasma supernatant (LCP Sup) at a 1:10 concentration, and negative supernatant (Neg Sup) as normal cells. The box plot graph depicts the median ± SD (*N* = 5). * *p* < 0.05 indicates statistical significance (non-parametric Kruskal–Wallis and Dunn’s multiple comparisons tests vs. cell control).

The expressions of ISGs, *RyDEN*, *IFITM-1*, *OAS-1*, *ISG15*, *ISG20*, *RSAD2*, and *MxA* were evaluated in A549 cells treated with IFN-α (100 units), CpG Sup 1:10, LCP Sup 1:10, or Neg Sup 1:10 for 24 h. Compared with untreated A549 cells, treatment with IFN-α (100 Units), CpG Sup 1:10, and LCP Sup 1:10 increased ISGs’ expression of *IFITM-1*, *ISG15*, *ISG20*, *MxA*, *OAS-1*, *RSAD2*, and *RyDEN* genes (Figure 2). Despite this increase, non-parametric Dunn’s multiple comparisons test suggested that only *MxA* showed a significant increase (*p* < 0.05) in A549 cells after 24 h of LCP Sup 1:10 treatment (Table 1).

### 2.3. Analysis of Cytokines, Chemokines, and Growth Factors in PBMC Supernatant

**Figure 3:**
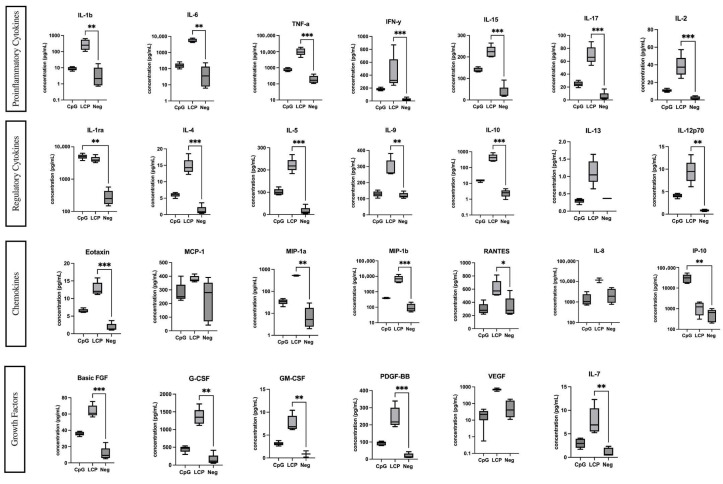
Analysis of cytokines, chemokines, and growth factors in PBMC supernatant using the multiplex BioPlex Pro Human Cytokine 27-plex assay kit. The experiment design contained CpG supernatant (CpG Sup) as an inducer for innate immunity, *Lactococcus lactis* strain Plasma supernatant (LCP) at different concentrations, and negative supernatant (Neg) as normal cells. The box plot graph depicts the median ± SD (*N* = 5). * *p* < 0.05, ** *p* < 0.01, *** *p* < 0.001 indicates statistical significance (non-parametric Kruskal–Wallis and Dunn’s multiple comparisons tests vs. cell control).

Cytokines, chemokines, and growth factors secreted by PBMCs were analyzed following 24 h of treatment with either the culture medium alone (negative control; Neg), 1 μM CpG ODN 2216 (positive control; CpG), or LC-Plasma (LCP). Twenty-seven cytokines, chemokines, and growth factors were analyzed, and we found no significant differences in IL-13, IL-8, MCP-1 and VEGF levels were induced by LC-Plasma and CpG stimulation. The other factors were significantly induced by LC-Plasma (Figure 3). In addition, IFN-α was previously confirmed to be significantly induced by LC-Plasma and CpG treatments under the same conditions [14] and also in a mouse pDC experiment in vivo [9].

## 3. Discussion

Airborne viruses, including H1N1 and SARS-CoV-2, remain significant contributors to upper respiratory infections. Despite the availability of vaccines against these infections, there remains a continuing need for better and more broadly preventive measures against these pathogens. Sarvepalli et al. also reviewed the challenges of directly acting antiviral (DAA) therapies, although they offer several advantages. The efficacy of these DAA therapies is compromised by factors such as high mutation rates, emerging new viruses, development of resistance, and narrow-spectrum effectiveness [17]. In addition, they presented a new perspective on various host-focused therapeutic approaches, including protein folding and maturation inhibitors, immunomodulators, cell therapies, antisense oligonucleotides, and others (such as convalescent plasma and repurposed drugs) [17]. LC-Plasma is classified as an immunomodulator, and in the current and our previous study [14], it demonstrated antiviral effects against different virus types (respiratory and mosquito-borne viruses) through increased production of IFN-α and upregulation of antiviral mRNAs.

It is well known that plasmacytoid pDCs are a subset of immune cells specialized in recognizing viral and bacterial nucleic acids, leading to the production of IFN-α and the priming of both innate and adaptive immune responses [18]. Synthetic TLR ligands, as well as pathogenic bacteria and viruses, are well-known activators of pDCs [19]. Recently, paraprobiotics have also been reported to have unique potency, similar to probiotics, and are recognized as bio-response modifiers (e.g., modulation of immunity, anti-inflammatory, and antiproliferative properties) [20]. LC-Plasma has been shown to activate pDCs within PBMCs and increase the production of IFN-α and humoral factors. As a result, LC-Plasma significantly upregulated IFN-stimulated genes (ISGs) in Huh-7 cells, leading to broad-spectrum antiviral effects against tropical viral infections such as Dengue virus (DENV), Chikungunya virus (CHIKV), and Zika virus (ZIKV) [14].

In the present study, we investigated the antiviral effects of LC-Plasma against H1N1 and SARS-CoV-2 using in vitro infection models. When comparing the antiviral effects of LC-Plasma against H1N1 and SARS-CoV-2, different cell lines had to be used due to the absence of ACE2 receptors in A549 cells. Therefore, the data suggests that the antiviral effect of LC-Plasma against H1N1 was considered relatively weaker than that against SARS-CoV-2, since approximately double the concentration of LC-Plasma supernatant was required to achieve the same antiviral effect (reduction in the RNA copy number) in H1N1 compared with the SARS-CoV-2 assay. The underlying mechanism is currently unknown. On one hand, it was reported that Type I IFN pretreatment of human airway epithelial cells reduced SARS-CoV-2 titers at much higher levels compared with H1N1, demonstrating differences in IFN sensitivities [21]. Alternatively, our previous results demonstrated that ISGs in Huh-7 cells were significantly upregulated by LC-Plasma-stimulated PBMC supernatant [14], exceeding the level of ISG upregulation observed in A549 cells in this study. This enhanced ISG response in Huh-7 cells may partly explain the more pronounced antiviral effect observed against SARS-CoV-2 compared with H1N1, implying that Huh-7 and A549 cells may exhibit inherent intrinsic differences in their innate immune responsiveness to interferons. For instance, Huh-7 cells may express higher levels of interferon-α/β receptors (IFNAR), leading to greater ISG induction upon exposure to LC-Plasma supernatant and thus stronger antiviral effects. However, further investigation is required to confirm this hypothesis.

In this study, a comprehensive analysis of cytokines, chemokines, and growth factors was carried out, showing several interesting points. Although the experimental protocol differs from in vivo conditions, LC-Plasma was in direct contact with all PBMCs, which included several kinds of lymphocytes simultaneously at high concentrations in the medium. Almost all measured parameters were increased by LCP Sup. Initially, it was hypothesized that LC-Plasma would be recognized specifically by pDCs and induce IFN-α production via pDC activation. However, this experiment involved the direct addition of LC-Plasma or CpG to PBMCs, a heterogeneous population of multiple immune cell types, revealing that LC-Plasma and CpG likely act not only on pDCs but also directly on other immune cells. Furthermore, secreted cytokines, chemokines, and growth factors may also act subsequently act immediately and directly on neighboring immune cells in a sort of positive feedback. In addition, direct cell-to-cell contact among immune cells may contribute to the observed response [22]. Therefore, the collective activation of the PBMC population likely led to increased production of multiple cytokines, chemokines, and growth factors. Although CpG treatment did not reach statistical significance, many factors showed a tendency toward increased production compared with the negative control, suggesting changes induced by the direct addition of these agents to the cells. More detailed mechanistic studies are necessary to confirm this speculation.

Our study nonetheless demonstrated that LC-Plasma increased IFN-α production via activation of pDCs within PBMCs. When IFN-α-containing supernatant was used to treat human cells, ISG expression was significantly upregulated. Comparing gene expression analyses from our previous study on Huh-7 cells [14] and current studies on A549 cells, *MxA* was identified as the most significantly upregulated ISG in response to LC-Plasma supernatant. This finding suggests that *MxA* may play a key role in mediating the broad-spectrum antiviral effects induced by humoral factors released from LC-Plasma-stimulated PBMCs. Mx proteins are conserved dynamin-like large GTPases, which localize to the nucleus when virus antigens fuse with the nuclear localization signal (NLS) at the C-terminal of MxA. The crystal structure of MxA suggests that MxA can recognize nucleoproteins (NP) [23] on viral ribonucleoproteins (vRNPs), forming MxA oligomers, also known as MxA rings, around the incoming vRNPs [24]. This action effectively prevents viral transcription by inhibiting vRNPs from entering the nucleus.

## 4. Materials and Methods

### 4.1. Participant Recruitment

Twenty-five healthy participants aged between 18 and 35 years old were recruited for the study. All participants were briefed on the study and gave written informed consent prior to providing whole blood. All enrolled participants had a normal heart rate (60–100 beats/min), body temperature (36.1–37.2 °C), and blood pressure (SBP < 120/DBP < 80 mmHg), as verified by a certified medical physician. The study was approved by the Universiti Malaya Medical Centre Medical Research Ethics Committee (MREC ID No. 202256-11216).

### 4.2. PBMCs’ Isolation, Preservation, and Culture

PBMCs were isolated from whole blood using CPT Vacutainer glass tubes (Becton Dickinson, CA, USA), according to the manufacturer’s protocol [25]. PBMCs were cryopreserved in Cellbanker2 (Zenogen Pharma, Fukushima, Japan) and stored at −80 °C. Upon thawing, PBMCs were cultured in RPMI-1640 media (Corning Inc., Corning, NY, USA) supplemented with 1% sodium pyruvate (1 mM) (Gibco, Thermo Fisher Scientific, Waltham, MA, USA), 0.25% 4-(2-hydroxyethyl)-1-piperazineethanesulfonic acid (HEPES) (2.5 mM) (Gibco, Thermo Fisher Scientific, Waltham, MA, USA), 1% penicillin/streptomycin (50 units/mL) (Gibco, Thermo Fisher Scientific, Waltham, MA, USA), 10% fetal bovine serum (FBS) (Gibco, Thermo Fisher Scientific, Waltham, MA, USA), and 1% non-essential amino acid (Gibco, Thermo Fisher Scientific, Waltham, MA, USA) [25].

### 4.3. Cells

A549 (adenocarcinoma human alveolar basal epithelial cells) (iCell-h011 strain, iCell Bioscience, Shanghai, China) cells were cultured and maintained in high-glucose (4 g/L) DMEM (Corning Inc., Corning, NY, USA). Vero CCL-81 cells (European Collection of Authenticated Cell Cultures, ECACC) were cultured in high-glucose DMEM containing 4 g/L glucose supplemented with 10% heat-inactivated FBS. Huh-7 cells (JCRB Cell Bank JCRB0403) were cultured and maintained in low-glucose (1 g/L) DMEM (Corning Inc., Corning, NY, USA) supplement with 10% heat-inactivated FBS. All cells were incubated at 37 °C and 5% CO_2_ [14].

### 4.4. Viruses

SARS-CoV-2 (SARS-CoV-2/MY/UM/6-3/2020, TIDREC; Malaysian human isolate confirmed by sequencing) was propagated and titrated in Vero cells, while the influenza virus (H1N1, A/Puerto Rico/8/1934) was propagated and titrated in MDCK cells (adult female cocker spaniel kidney) (iCell-c021, iCell Bioscience, Shanghai, China). The in vitro SARS-CoV-2 assays were conducted in the Biosafety Level 3 (BSL-3) biocontainment laboratory at TIDREC, Universiti Malaya. The FBS was reduced to 2% in all media used for virus infection assays.

### 4.5. Preparation of LC-Plasma Suspension

LC-Plasma was provided by Kirin Holdings Co., Ltd., (Fujisawa, Kanagawa, Japan), in heat-killed lyophilized form and was stored at 4 °C in the dark until use. Reconstitution of the LC-Plasma was carried out using RPMI-1640 and sonicated (Omni-Ruptor 4000, OMNI International, Kennesaw, GA, USA) for two minutes at 40 watts on ice [14]. A final concentration of 10 μg/mL LC-Plasma was used for PBMC stimulation. Fresh preparations of LC-Plasma were used for each round of stimulation.

### 4.6. PBMC Supernatant

Thawed PBMCs were used seeded at 2 × 10^5^ cells/mL in 96-well plates and stimulated with 10 μg/mL LC-Plasma for 24 h [14]. The representative TLR9 ligand, 1 μM CpG ODN 2216 (Invivogen, San Diego, CA, USA), was used as the positive control during the stimulation [26]. After 24 h, the PBMC supernatants from five random participants were pooled to achieve a total of five distinct pools amounting to five biological replicates (*N* = 5) for analysis. The supernatant was harvested by centrifugation, pooled, aliquoted, and stored at −80 °C until further use. Freeze-thawing of the supernatants was avoided.

### 4.7. Antiviral Assay

A549 cells were used as an in vitro infection model for the H1N1 antiviral study. However, Huh-7 cells were used to assess the anti-SARS-CoV-2 effects of LC-Plasma, as A549 cells lack ACE2 receptors, the entry receptors for SARS-CoV-2. Huh-7 cells and A549 cells were seeded in 24-well plates at 1 × 10^5^ cells/well overnight. The cells were then incubated with serially diluted PBMC supernatant for 24 h before virus infection. In total, 100 units of recombinant universal Type I IFN-α (PBL assay science, Piscataway, NJ, USA) was used as a positive control [14]. Thereafter, virus inoculum at a multiplicity of infection (MOI) of 0.1 was added to the cells and preabsorbed for one hour at 37 °C. After 48 h post-infection, the supernatants were harvested, aliquoted, and stored at −80 °C for virus titer quantitation by qRT-PCR. The abbreviations for each treatment group and their corresponding supernatant dilutions derived from PBMCs are as follows: VC, untreated Huh-7 or A549 cells infected with the virus at a MOI of 0.1 only; Neg Sup, negative control, cells treated with the supernatant of unstimulated PBMCs; CpG Sup, diluted supernatant derived from 1 μM of CpG ODN 2216-stimulated PBMCs; LCP Sup, diluted supernatant of 10 μg/mL LC-Plasma-stimulated PBMCs. Dilutions are stated post-fixing. They are described in full in Supplementary Table S1.

### 4.8. Quantitative RT-PCR of H1N1 and SARS-CoV-2

Viral RNA from the supernatants was extracted using the QIAampR viral RNA extraction kit (QIAGEN, Venlo, The Netherlands, QIAG-52904) following the manufacturer’s protocol. The extracted RNA was reverse-transcribed and amplified using the SensiFast SYBER Hi-ROX one-step kit (Bioline, London, UK, BIO-73005) on the StepOnePlus Real-Time PCR System. The primers used were H1N1 NA gene (forward: GCAGCTGTGGTCCAGTGTAT; reverse: GCCACAACATCTTGCCTCAC) and SARS-CoV-2 S gene (forward: CGGCCTTACTGTTTTGCCAC, reverse: TGTACCCGCTAACAGTGCAG).

### 4.9. Gene Expression Study in Stimulated A549 Cells

A549 cells were stimulated with 10-fold serially diluted PBMC supernatant for 24 h. Total cellular RNA was then extracted using the RNeasy Kit (QIAGEN, Venlo, The Netherlands, QIAG-74106), and cDNA synthesis was carried out using the iScript cDNA synthesis kit (BioRad, Hercules, CA, USA, 1708891) following the manufacturer’s protocol. The differential ISGs’ expression levels were determined using the comparative Ct (2^−ΔΔCt^) [27] method. qRT-PCR was carried out using the SYBR green-based Luna^®^ Universal qRT-PCR Master Mix (NEB, Ipswich, MA, USA, M3003L) on the QuantStudio™ 5 Real-Time PCR System (Applied Biosystems, Foster City, CA, USA). The β-actin (ACTB) gene was used for normalization. The gene expression levels were referenced to the untreated A549 cells (cell control). The ISGs examined were *RyDEN*, *IFITM-1*, *OAS-1*, *ISG15*, *ISG20*, *RSAD2*, and *MxA*, and the primers used were adapted from a previous publication [26].

### 4.10. Cytokine, Chemokine, and Growth Factor Analysis in Stimulated PBMCs

PBMCs were treated with the culture medium only (Neg, negative control), 1 μM CpG ODN 2216 (CpG, positive control), or LC-Plasma (LCP) for 24 h. The resulting supernatants were collected, and the released cytokines, chemokines and growth factors were analyzed using the Bio-Plex Pro Human Cytokine 27-plex Assay (BioRad, Hercules, CA, USA, M500KCAF0Y) with a Luminex 200 Instrument system (Luminex, Austin, TX, USA) according to the manufacturer’s protocols. Twenty-seven analytes were examined: cytokines—IL-1β, IL-6, TNF-α, IFN-γ, IL-15, IL-17, IL-2, IL-1ra, IL-4, IL-5, IL-9, IL-10, IL-13, IL-12p70, and IL-7; chemokines—eotaxin, MCP-1, MIP-1a, MIP-1b, RANTES, IL-8, and IP-10; and growth factors—FGF, G-CSF, GM-CSF, PDGF-BB, and VEGF.

### 4.11. Statistical Analysis

All data are presented as the median ± SD from five biological replicates (*N* = 5). Data were analyzed using the non-parametric Kruskal–Wallis and Dunn’s multiple comparisons test using GraphPad Prism 10 software (GraphPad Software Inc., San Diego, CA, USA).

## 5. Conclusions

In summary, our findings, together with previous studies, suggest that enhanced IFN-α production, including other humoral factors stimulation leading to upregulation of ISGs’ (*MxA*) expression, is a possible mechanism contributing to LC-Plasma’s antiviral activity against H1N1 and SARS-CoV-2.

## Figures and Tables

**Table 1:** Fold increase in ISGs of A549 cells following treatment with recombinant IFN-α (100 units), CpG Sup 1:10, and LCP Sup 1:10 (median ± SD, *N* = 5).

ISGs	Fold Increase in ISGs in A549			
	IFN-α (100 U)	CpG 1:10	LCP 1:10	Neg 1:10
*IFITM-1*	7.06 ± 1.91	5.30 ± 2.64	1.75 ± 0.88	0.67 ± 0.53
*ISG15*	94.50 ± 78.80	57.67 ± 85.45	7.14 ± 17.26	1.16 ± 0.74
*ISG20*	3.55 ± 1.92	3.35 ± 2.11	2.00 ± 2.20	1.19 ± 1.28
*MxA*	2442.00 ± 1243.00	1441.00 ± 1253.00	96.04 ± 70.30 *	1.12 ± 0.77
*OAS-1*	6.57 ± 31.02	8.42 ± 28.68	4.82 ± 13.35	1.15 ± 1.08
*RSAD2*	239.60 ± 136.90	89.41 ± 122.10	12.52 ± 15.44	0.96 ± 1.77
*RYDEN*	5.09 ± 2.60	8.89 ± 6.85	2.37 ± 3.50	2.00 ± 5.37

*—indicates *p* < 0.05.

## Data Availability

The original contributions presented in this study are included in the article/Supplementary Materials. Further inquiries can be directed to the corresponding authors.

## Supplementary Materials

The following supporting information can be downloaded at https://www.mdpi.com/article/10.3390/ijms262311573/s1.

## Abbreviations

The following abbreviations are used in this manuscript.

CHIKV	Chikungunya virus
COVID-19	Coronavirus disease 2019
CpG	CpG ODN 2216
DAA	Directly acting antiviral
DCs	Dendritic cells
DENV	Dengue virus
ECACC	European Collection of Authenticated Cell Cultures
FBS	Fetal bovine serum
H1N1	Influenza A virus subtype H1N1
HDT	Host-directed therapy
HEPES	4-(2-hydroxyethyl)-1-piperazineethanesulfonic acid
HSC	Hematopoietic stem cells
IAV	Influenza A virus
IFN-α	Interferon-α
IFNAR	Interferon-α/β receptors
ISGs	Interferon-stimulated genes
LC-Plasma	*Lactococcus lactis* strain plasma
LCP Sup	Supernatant derived from LC-Plasma-stimulated PBMCs
mDCs	Myeloid dendritic cells
MOI	Multiplicity of infection
mRNAs	Messenger RNA
NP	Nucleoproteins
PBMCs	Peripheral blood mononuclear cells
pDCs	Plasmacytoid dendritic cells
SARS-CoV-2	Severe acute respiratory syndrome coronavirus 2
TLR	Toll-like receptor
vRNPs	Viral ribonucleoproteins
ZIKV	Zika virus

## References

[1] Variants of the Coronavirus SARS-CoV-2|RIVM. https://www.rivm.nl/en/coronavirus-covid-19/current/variants.

[2] Das S., Samanta S., Banerjee J., Pal A., Giri B., Kar S.S., Dash S.K. (2022). Is Omicron the End of Pandemic or Start of a New Innings?. Travel Med. Infect. Dis..

[3] Keyel A.C., Russell A., Plitnick J., Rowlands J.V., Lamson D.M., Rosenberg E., George K.S. (2022). SARS-CoV-2 Vaccine Breakthrough by Omicron and Delta Variants, New York, USA. Emerg. Infect. Dis..

[4] Franco-Luiz A.P.M., Fernandes N.M.G.S., Silva T.B.d.S., Bernardes W.P.d.O.S., Westin M.R., Santos T.G., Fernandes G.d.R., Simões T.C., Silva E.F.E., Gava S.G. (2023). Longitudinal Study of Humoral Immunity against SARS-CoV-2 of Health Professionals in Brazil: The Impact of Booster Dose and Reinfection on Antibody Dynamics. Front. Immunol..

[5] Gao F.X., Wu R.X., Shen M.Y., Huang J.J., Li T.T., Hu C., Luo F.Y., Song S.Y., Mu S., Hao Y.N. (2022). Extended SARS-CoV-2 RBD Booster Vaccination Induces Humoral and Cellular Immune Tolerance in Mice. iScience.

[6] Uversky V.N., Redwan E.M., Makis W., Rubio-Casillas A. (2023). IgG4 Antibodies Induced by Repeated Vaccination May Generate Immune Tolerance to the SARS-CoV-2 Spike Protein. Vaccines.

[7] Blümel J., Burger R., Drosten C., Gröner A., Gürtler L., Heiden M., Hildebrandt M., Jansen B., Klamm H., Montag-Lessing T. (2009). Influenza Virus. Transfus. Med. Hemotherapy.

[8] Liu J.-W., Lin S.-H., Wang L.-C., Chiu H.-Y., Lee J.-A. (2021). Comparison of Antiviral Agents for Seasonal Influenza Outcomes in Healthy Adults and Children: A Systematic Review and Network Meta-Analysis. JAMA Netw. Open.

[9] Jounai K., Ikado K., Sugimura T., Ano Y., Braun J., Fujiwara D. (2012). Spherical Lactic Acid Bacteria Activate Plasmacytoid Dendritic Cells Immunomodulatory Function via Tlr9-Dependent Crosstalk with Myeloid Dendritic Cells. PLoS ONE.

[10] Cho C.H., Yoon S.Y., Lee C.K., Lim C.S., Cho Y. (2015). Effect of Interleukin-29 on Interferon-α Secretion by Peripheral Blood Mononuclear Cells. Cell J..

[11] Ye Y., Gaugler B., Mohty M., Malard F. (2020). Plasmacytoid Dendritic Cell Biology and Its Role in Immune-Mediated Diseases. Clin. Transl. Immunol..

[12] Schuster P., Donhauser N., Pritschet K., Ries M., Haupt S., Kittan N.A., Korn K., Schmidt B. (2010). Co-Ordinated Regulation of Plasmacytoid Dendritic Cell Surface Receptors upon Stimulation with Herpes Simplex Virus Type 1. Immunology.

[13] Honda K., Ohba Y., Yanai H., Hegishi H., Mizutani T., Takaoka A., Taya C., Taniguchi T. (2005). Spatiotemporal Regulation of MyD88-IRF-7 Signalling for Robust Type-I Interferon Induction. Nature.

[14] Low Z.X., Kanauchi O., Tiong V., Sahimin N., Lani R., Tsuji R., AbuBakar S., Hassandarvish P. (2024). The Antiviral Effects of Heat-Killed Lactococcus Lactis Strain Plasma Against Dengue, Chikungunya, and Zika Viruses in Humans by Upregulating the IFN-α Signaling Pathway. Microorganisms.

[15] Kaufmann S.H.E., Dorhoi A., Hotchkiss R.S., Bartenschlager R. (2018). Host-Directed Therapies for Bacterial and Viral Infections. Nat. Rev. Drug Discov..

[16] Tian W.-J., Wang X.-J. (2023). Broad-Spectrum Antivirals Derived from Natural Products. Viruses.

[17] Sarvepalli S., Vadarevu S. (2025). Non-Antiviral Therapies for Viral Infections: Harnessing Host Mechanisms. Int. Immunopharmacol..

[18] Manz M.G. (2018). Plasmacytoid Dendritic Cells: Origin Matters. Nat. Immunol..

[19] Libri N.A., Barker S.J., Rosenberg W.M.C., Semper A.E. (2009). A Class C CpG Toll-like Receptor 9 Agonist Successfully Induces Robust Interferon-Alpha Production by Plasmacytoid Dendritic Cells from Patients Chronically Infected with Hepatitis C. J. Viral Hepat..

[20] Akter S., Park J.-H., Jung H.K. (2020). Potential Health-Promoting Benefits of Paraprobiotics, Inactivated Probiotic Cells. J. Microbiol. Biotechnol..

[21] Lokugamage K.G., Hage A., de Vries M., Valero-Jimenez A.M., Schindewolf C., Dittmann M., Rajsbaum R., Menachery V.D. (2020). Type I Interferon Susceptibility Distinguishes SARS-CoV-2 from SARS-CoV. J. Virol..

[22] Hwang I. (2013). Cell-Cell Communication Via Extracellular Membrane Vesicles and Its Role in the Immune Response. Mol. Cells.

[23] Wisskirchen C., Ludersdorfer T.H., Müller D.A., Moritz E., Pavlovic J. (2011). Interferon-Induced Antiviral Protein MxA Interacts with the Cellular RNA Helicases UAP56 and URH49. J. Biol. Chem..

[24] Sadler A.J., Williams B.R.G. (2008). Interferon-Inducible Antiviral Effectors. Nat. Rev. Immunol..

[25] Low Z.X., Kanauchi O., AbuBakar S., Tiong V., Hassandarvish P. (2025). Protocol for Screening Host-Targeting Antivirals (HTAs) Using Human PBMCs and pDCs. Bio-protocol.

[26] Tsuji R., Yamamoto N., Yamada S., Fujii T., Yamamoto N., Kanauchi O. (2018). Induction of Anti-Viral Genes Mediated by Humoral Factors upon Stimulation with Lactococcus Lactis Strain Plasma Results in Repression of Dengue Virus Replication in Vitro. Antivir. Res..

[27] Sainz B., Tencate V., Uprichard S.L. (2009). Three-Dimensional Huh7 Cell Culture System for the Study of Hepatitis C Virus Infection. Virol. J..

