# Supplementary material for: Stimulation of Peripheral Blood Mononuclear Cells with Lactococcus lactis Strain Plasma Elicits Antiviral Effects Against H1N1 and SARS-CoV-2
Source: Int J Mol Sci. 2025 Nov 28;26(23):11573. doi: 10.3390/ijms262311573 (PMC12691877; doi:10.3390/ijms262311573)
Supplement: Supplementary file 1 [file ijms-26-11573-s001.zip › ijms-3963100-supplementary.pdf]

# *Supplementary Material*

## 1 Supplementary Table

**Table S1.** Treatment groups and their corresponding supernatant dilutions derived from PBMCs

| Treatment Groups (Short forms) | Description |
|---|---|
| VC | Virus control refers to untreated Huh-7 or A549 cells infected with virus at an MOI of 0.1. |
| Neg Sup 1:10 | 10-fold diluted supernatant derived from PBMCs (without treatment, Negative control) |
| CpG Sup 1:10 | 10-fold diluted supernatant derived from 1 µM of CpG ODN 2216-stimulated PBMCs |
| LCP Sup 1:10 | 10-fold diluted supernatant derived from 10 µg/mL of LC-Plasma-stimulated PBMCs |
| LCP Sup 1:100 | 100-fold diluted supernatant derived from 10 µg/mL of LC-Plasma-stimulated PBMCs |
| LCP Sup 1:1000 | 1000-fold diluted supernatant derived from 10 µg/mL of LC-Plasma-stimulated PBMCs |
| LCP Sup 1:5 | 5-fold diluted supernatant derived from 10 µg/mL of LC-Plasma-stimulated PBMCs |
| LCP Sup 1:15 | 15-fold diluted supernatant derived from 10 µg/mL of LC-Plasma-stimulated PBMCs |
